# 2-[(4-Benzhydrylpipérazin-1-yl)méthyl]acrylonitrile

**DOI:** 10.1107/S1600536808025403

**Published:** 2008-09-06

**Authors:** Fatma Ben Amor, Mohamed Ould M’hamed, Hédi Mrabet, Ahmed Driss, Mohamed Lotfi Efrit

**Affiliations:** a Laboratoire de Matériaux et Cristallochimie, Faculté des Sciences de Tunis, Université de Tunis El Manar, 2092 El Manar I Tunis, Tunisie; b Laboratoire de Synthèse Organique et Hétérocyclique, Faculté des Sciences de Tunis, Université de Tunis El Manar, 2092 El Manar I Tunis, Tunisie

## Abstract

In the title compound, 2-[(4-benz­hydryl­piperazin-1-yl)­methyl]­acrylo­nitrile, C_21_H_23_N_3_, the substituted piperazine ring adopts a chair conformation and the dihedral angle between the mean planes of the aromatic rings is 71.61 (8)°.

## Littérature associée

Pour littérature associée, voir: Mikami *et al.* (1991 ▶); Mrabet & Zantour (2004 ▶); Ould M’hamed *et al.* (2007 ▶, 2008 ▶); Toumi *et al.* (2007 ▶).
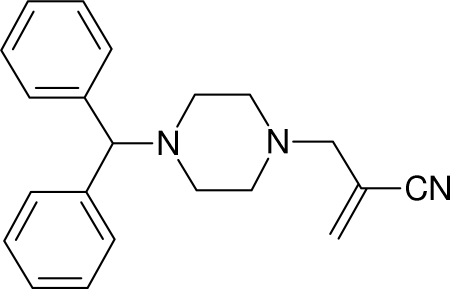


## Partie expérimentale

### 

#### Données crystallines

C_21_H_23_N_3_
*M* *_r_* = 317.42
Monoclinique, 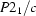

*a* = 17.443 (5) Å
*b* = 6.183 (3) Å
*c* = 17.717 (5) Å
β = 107.00 (3)°
*V* = 1827.4 (12) Å^3^
*Z* = 4
Radiation Mo *K*α
μ = 0.07 mm^−1^
*T* = 298 (2) K
0.72 × 0.58 × 0.22 mm

#### Collection des données

Diffractomètre Enraf–Nonius CAD-4
Correction d’absorption: ψ scan (North *et al.*, 1968 ▶) *T*_min_ = 0.904, *T*_max_ = 0.985
3708 réflexions mesurées
3587 réflexions independantes
1625 réflexions avec *I* > 2σ(*I*)
*R*_int_ = 0.052
2 réflexions de référence fréquence: 120 min déclin d’intensité: 7%

#### Affinement

*R*[*F* ^2^ > 2σ(*F* ^2^)] = 0.054
*wR*(*F* ^2^) = 0.156
*S* = 0.97
3587 réflexions
309 paramètres
Tous les paramètres des atomes H affinés
Δρ_max_ = 0.17 e Å^−3^
Δρ_min_ = −0.20 e Å^−3^

### 

Collection des données: *CAD-4 EXPRESS* (Enraf–Nonius, 1994 ▶); affinement des paramètres de la maille: *CAD-4 EXPRESS*; réduction des données: *XCAD4* (Harms & Wocadlo, 1995 ▶); programme(s) pour la solution de la structure: *SHELXS97* (Sheldrick, 2008 ▶); programme(s) pour l’affinement de la structure: *SHELXL97* (Sheldrick, 2008 ▶); graphisme moléculaire: *DIAMOND* (Brandenburg, 1998 ▶); logiciel utilisé pour préparer le matériel pour publication: *SHELXL97*.

## Supplementary Material

Crystal structure: contains datablocks global, I. DOI: 10.1107/S1600536808025403/hb2772sup1.cif

Structure factors: contains datablocks I. DOI: 10.1107/S1600536808025403/hb2772Isup2.hkl

Additional supplementary materials:  crystallographic information; 3D view; checkCIF report

## Figures and Tables

**Fig. 1.**
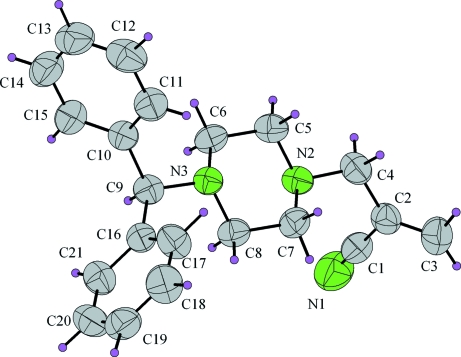
Représentation de la molécule C21H23N3. Les ellipsoïdes d'agitation thermique ont 50% de probabilité de présence.

## supplementary crystallographic information

### Comment

Des travaux récents réalisés dans notre laboratoire ont montré que les dérivés acryliques sont d'excellents agents bi-électrophiles-1,3. Ils constituent des précurseurs d'une large gamme d'hétérocycles (Mrabet & Zantour, 2004; Ould M'hamed *et al.*, 2007). Ces synthons sont également de bons diénophiles dans la réaction de Diels–Alder (Ould M'hamed *et al.*, 2008), ce qui permet d'obtenir des composés bicycliques très recherchés en tant qu'analogues de substances naturelles (Mikami *et al.*, 1991).

La molécule C_21_H_23_N_3_ est caractérisée par la présence d'un groupement pipérazinyle ayant chaque atome d'azote substitué. L'atome d'azote N3 est lié au carbone C9 qui est lui-même lié à deux noyaux phényles. L'autre atome d'azote N2 est lié au carbone C4 qui porte le groupement 1-cyanovinyle (C1, C2, C3, N1).

Le noyau phényle formé par les atomes C10, C11, C12, C13, C14 et C15 (phényle 1) est sur un plan moyen d'équation: 12,26 (1) *x* - 2,226 (7) *y* - 14,04 (1) *z* = 0,493 (8).

La déviation moyenne de ses atomes est de 0,002 Å. Les atomes de carbone C16, C17, C18, C19, C20 et C21 du deuxième phényle (phényle 2) définissent un plan moyen d'équation 13,76 (1) *x* - 1,692 (8) *y* + 5,23 (2) *z* = 4,399 (4). Ils dévient en moyenne de ce plan de 0,007 Å. L'atome de carbone C9 qui porte les deux phényles dévie de leurs plans de 0,037 (4) Å et 0,073 (4) Å respectivement. Les deux phényles forment entre eux un angle de 71,61 (8)°. Les atomes N1, C1, C2 et C3 du groupement 1-cyanovinyle forment eux aussi un plan d'équation: -11,20 (4) *x* + 2,303 (9) *y* + 14,68 (4) *z* = 4,062 (6). Leur déviation moyenne est de 0,0006 Å. Ce groupement fait un angle de 4,8(3)° avec le premier phényle et un angle de 76,1(2)° avec le deuxième phényle. La valeur de la distance N1—C1 [1,139 (3) Å] et les valeurs moyennes des distances N—C [1,469 (3) Å] et C—C [1,518 (4) Å] du groupement pipérazinyle sont en accord avec celles rencontrées dans des composés ayant ces types de liaisons (Toumi *et al.*,  2007).

### Experimental

La réaction est effectuée dans un ballon de 250 ml muni d'un barreau aimanté surmonté d'un séparateur à eau et d'un réfrigérant ascendant. Le reflux est réalisé à l'aide d'un bain d'huile. La réaction est terminée lorsqu'il ne se forme plus d'eau (environ 2 heures). A une solution d'acide cyanoacétique (0,3 mol) dans 300 ml de benzène, sont ajoutés 0,72 mol de paraformaldéhyde et 0,3 mol de 1-(diphénylméthyl)pipérazine. Le mélange est chauffé à reflux pendant 4 heures. Après refroidissement, le solvant est évaporé sous sec et le résidu est repris par 80 ml de chloroforme, lavé à l'eau (2 × 20 ml) et séché sur du sulfate de magnésium. Le solvant est évaporé sous sec et le produit obtenu est purifié par chromatographie sur colonne en utilisant l'éther diéthylique comme éluant.

### Refinement

[Please give range of refined C—H distances]

### Crystal data

**Table:**

C_21_H_23_N_3_	*F*(000) = 680
*M**_r_* = 317.42	*D*_x_ = 1.154 Mg m^−^^3^
Monoclinic, *P*2_1_/*c*	Mo *K*α radiation, λ = 0.71073 Å
Hall symbol: -P 2ybc	Cell parameters from 25 reflections
*a* = 17.443 (5) Å	θ = 11.6–15.0°
*b* = 6.183 (3) Å	µ = 0.07 mm^−^^1^
*c* = 17.717 (5) Å	*T* = 298 K
β = 107.00 (3)°	Slab, colourless
*V* = 1827.4 (12) Å^3^	0.72 × 0.58 × 0.22 mm
*Z* = 4	

### Data collection

**Table:**

Enraf–Nonius CAD-4 diffractometer	1625 reflections with *I* > 2σ(*I*)
Radiation source: fine-focus sealed tube	*R*_int_ = 0.052
graphite	θ_max_ = 26.0°, θ_min_ = 2.4°
Non–profiled ω/2θ scans	*h* = −21→1
Absorption correction: ψ scan (North *et al.*, 1968)	*k* = 0→7
*T*_min_ = 0.904, *T*_max_ = 0.985	*l* = −21→21
3708 measured reflections	2 standard reflections every 120 min
3587 independent reflections	intensity decay: 7%

### Refinement

**Table:**

Refinement on *F*^2^	Primary atom site location: structure-invariant direct methods
Least-squares matrix: full	Secondary atom site location: difference Fourier map
*R*[*F*^2^ > 2σ(*F*^2^)] = 0.054	Hydrogen site location: inferred from neighbouring sites
*wR*(*F*^2^) = 0.156	All H-atom parameters refined
*S* = 0.98	*w* = 1/[σ^2^(*F*_o_^2^) + (0.0682*P*)^2^] where *P* = (*F*_o_^2^ + 2*F*_c_^2^)/3
3587 reflections	(Δ/σ)_max_ < 0.001
309 parameters	Δρ_max_ = 0.17 e Å^−^^3^
0 restraints	Δρ_min_ = −0.20 e Å^−^^3^

### Special details

**Table:**

Geometry. All e.s.d.'s (except the e.s.d. in the dihedral angle between two l.s. planes) are estimated using the full covariance matrix. The cell e.s.d.'s are taken into account individually in the estimation of e.s.d.'s in distances, angles and torsion angles; correlations between e.s.d.'s in cell parameters are only used when they are defined by crystal symmetry. An approximate (isotropic) treatment of cell e.s.d.'s is used for estimating e.s.d.'s involving l.s. planes.
Refinement. Refinement of *F*^2^ against ALL reflections. The weighted *R*-factor *wR* and goodness of fit *S* are based on *F*^2^, conventional *R*-factors *R* are based on *F*, with *F* set to zero for negative *F*^2^. The threshold expression of *F*^2^ > σ(*F*^2^) is used only for calculating *R*-factors(gt) *etc*. and is not relevant to the choice of reflections for refinement. *R*-factors based on *F*^2^ are statistically about twice as large as those based on *F*, and *R*- factors based on ALL data will be even larger.

### Fractional atomic coordinates and isotropic or equivalent isotropic displacement parameters (Å^2^)

**Table:**

	*x*	*y*	*z*	*U*_iso_*/*U*_eq_	
N1	−0.13498 (16)	0.4548 (5)	0.10237 (17)	0.0949 (9)	
N2	0.08178 (11)	0.5724 (3)	0.18358 (11)	0.0514 (5)	
N3	0.22797 (11)	0.3529 (3)	0.17780 (11)	0.0484 (5)	
C1	−0.09372 (17)	0.6013 (5)	0.11096 (15)	0.0625 (7)	
C2	−0.04111 (15)	0.7881 (4)	0.12169 (16)	0.0564 (7)	
C3	−0.0645 (2)	0.9613 (6)	0.0767 (2)	0.0792 (9)	
C4	0.03676 (16)	0.7713 (5)	0.18724 (18)	0.0616 (7)	
C5	0.15513 (15)	0.5667 (5)	0.25155 (16)	0.0558 (7)	
C6	0.20291 (16)	0.3636 (5)	0.24995 (15)	0.0535 (7)	
C7	0.10589 (17)	0.5601 (5)	0.11123 (16)	0.0560 (7)	
C8	0.15533 (15)	0.3575 (5)	0.11019 (15)	0.0545 (7)	
C9	0.27587 (14)	0.1535 (4)	0.17883 (14)	0.0492 (6)	
C10	0.35248 (13)	0.1564 (4)	0.24803 (14)	0.0504 (6)	
C11	0.40273 (16)	0.3359 (5)	0.26327 (17)	0.0642 (8)	
C12	0.47357 (17)	0.3339 (7)	0.3254 (2)	0.0781 (10)	
C13	0.4938 (2)	0.1530 (7)	0.3721 (2)	0.0842 (11)	
C14	0.4453 (2)	−0.0264 (7)	0.3578 (2)	0.0816 (10)	
C15	0.37478 (18)	−0.0246 (6)	0.29607 (17)	0.0660 (8)	
C16	0.29580 (13)	0.1168 (4)	0.10145 (14)	0.0495 (6)	
C17	0.32827 (19)	0.2798 (5)	0.06644 (17)	0.0685 (8)	
C18	0.3497 (2)	0.2389 (6)	−0.00144 (19)	0.0807 (10)	
C19	0.33888 (18)	0.0384 (6)	−0.03670 (18)	0.0738 (9)	
C20	0.30501 (19)	−0.1234 (6)	−0.00327 (19)	0.0746 (9)	
C21	0.28447 (16)	−0.0861 (5)	0.06555 (18)	0.0623 (7)	
H13	−0.0285 (19)	1.088 (5)	0.0898 (18)	0.098 (11)*	
H23	−0.1189 (16)	0.972 (4)	0.0391 (16)	0.068 (8)*	
H14	0.0235 (14)	0.767 (4)	0.2404 (16)	0.074 (8)*	
H24	0.0693 (16)	0.909 (4)	0.1835 (15)	0.075 (8)*	
H15	0.1394 (13)	0.573 (4)	0.2995 (14)	0.058 (7)*	
H25	0.1883 (16)	0.704 (4)	0.2488 (15)	0.078 (8)*	
H16	0.1723 (15)	0.231 (4)	0.2543 (14)	0.068 (8)*	
H26	0.2498 (14)	0.361 (3)	0.2972 (13)	0.053 (6)*	
H17	0.0579 (14)	0.552 (4)	0.0641 (15)	0.057 (7)*	
H27	0.1379 (14)	0.692 (4)	0.1039 (13)	0.060 (7)*	
H18	0.1224 (15)	0.228 (4)	0.1109 (14)	0.068 (8)*	
H28	0.1719 (13)	0.360 (4)	0.0624 (14)	0.055 (7)*	
H19	0.2430 (12)	0.032 (3)	0.1842 (11)	0.039 (6)*	
H111	0.3885 (13)	0.460 (4)	0.2327 (14)	0.056 (8)*	
H112	0.5043 (17)	0.462 (5)	0.3322 (17)	0.088 (10)*	
H113	0.5413 (19)	0.155 (5)	0.4162 (18)	0.093 (10)*	
H114	0.4583 (19)	−0.147 (5)	0.3893 (18)	0.096 (11)*	
H115	0.3425 (16)	−0.147 (5)	0.2845 (15)	0.078 (10)*	
H117	0.3375 (17)	0.426 (5)	0.0924 (18)	0.094 (10)*	
H118	0.3684 (18)	0.350 (5)	−0.0223 (17)	0.086 (10)*	
H119	0.3571 (17)	0.006 (5)	−0.0838 (19)	0.100 (10)*	
H120	0.2960 (16)	−0.263 (5)	−0.0260 (17)	0.084 (9)*	
H121	0.2654 (16)	−0.199 (4)	0.0925 (15)	0.072 (9)*	

### Atomic displacement parameters (Å^2^)

**Table:**

	*U*^11^	*U*^22^	*U*^33^	*U*^12^	*U*^13^	*U*^23^
N1	0.0769 (18)	0.088 (2)	0.102 (2)	−0.0164 (16)	−0.0001 (16)	0.0096 (17)
N2	0.0447 (11)	0.0591 (14)	0.0510 (11)	−0.0024 (10)	0.0149 (9)	−0.0069 (10)
N3	0.0423 (11)	0.0535 (13)	0.0495 (11)	−0.0019 (10)	0.0135 (9)	−0.0029 (10)
C1	0.0554 (16)	0.067 (2)	0.0588 (16)	0.0055 (16)	0.0067 (14)	0.0023 (14)
C2	0.0517 (15)	0.0572 (17)	0.0614 (15)	0.0058 (14)	0.0182 (13)	−0.0068 (14)
C3	0.077 (2)	0.067 (2)	0.089 (2)	0.0144 (19)	0.016 (2)	0.0031 (19)
C4	0.0583 (17)	0.0565 (18)	0.0700 (18)	0.0005 (15)	0.0187 (14)	−0.0111 (15)
C5	0.0483 (14)	0.0703 (19)	0.0507 (15)	−0.0033 (14)	0.0173 (13)	−0.0077 (14)
C6	0.0451 (14)	0.0679 (18)	0.0462 (14)	−0.0038 (14)	0.0116 (12)	0.0029 (13)
C7	0.0476 (14)	0.0635 (19)	0.0549 (16)	0.0044 (14)	0.0116 (13)	0.0034 (14)
C8	0.0485 (15)	0.0639 (19)	0.0496 (15)	−0.0018 (15)	0.0118 (12)	−0.0023 (13)
C9	0.0439 (13)	0.0488 (16)	0.0553 (15)	−0.0116 (13)	0.0151 (11)	0.0002 (12)
C10	0.0430 (13)	0.0553 (16)	0.0538 (14)	−0.0007 (13)	0.0156 (11)	−0.0040 (13)
C11	0.0516 (16)	0.066 (2)	0.0726 (19)	−0.0047 (15)	0.0145 (15)	−0.0038 (16)
C12	0.0488 (18)	0.094 (3)	0.089 (2)	−0.0114 (19)	0.0162 (17)	−0.029 (2)
C13	0.0546 (19)	0.115 (3)	0.073 (2)	0.021 (2)	0.0031 (17)	−0.019 (2)
C14	0.074 (2)	0.092 (3)	0.071 (2)	0.023 (2)	0.0090 (18)	0.007 (2)
C15	0.0605 (18)	0.068 (2)	0.0673 (19)	0.0052 (17)	0.0149 (15)	0.0057 (16)
C16	0.0382 (13)	0.0520 (16)	0.0557 (14)	−0.0013 (11)	0.0097 (11)	−0.0020 (12)
C17	0.086 (2)	0.0594 (19)	0.0687 (18)	−0.0062 (16)	0.0367 (16)	−0.0035 (15)
C18	0.098 (2)	0.079 (2)	0.078 (2)	0.002 (2)	0.0455 (19)	0.008 (2)
C19	0.0669 (19)	0.098 (3)	0.0570 (18)	0.0194 (18)	0.0184 (15)	−0.0029 (18)
C20	0.0671 (19)	0.075 (2)	0.077 (2)	0.0013 (18)	0.0152 (16)	−0.0247 (19)
C21	0.0546 (16)	0.0583 (19)	0.0751 (19)	−0.0043 (14)	0.0208 (14)	−0.0071 (16)

### Geometric parameters (Å, °)

**Table:**

N1—C1	1.139 (3)	C9—C10	1.527 (3)
N2—C7	1.463 (3)	C9—H19	0.97 (2)
N2—C4	1.471 (3)	C10—C11	1.391 (4)
N2—C5	1.479 (3)	C10—C15	1.391 (4)
N3—C8	1.467 (3)	C11—C12	1.394 (4)
N3—C6	1.469 (3)	C11—H111	0.93 (2)
N3—C9	1.486 (3)	C12—C13	1.374 (5)
C1—C2	1.453 (4)	C12—H112	0.95 (3)
C2—C3	1.326 (4)	C13—C14	1.373 (5)
C2—C4	1.511 (4)	C13—H113	0.96 (3)
C3—H13	0.99 (3)	C14—C15	1.387 (4)
C3—H23	0.99 (3)	C14—H114	0.92 (3)
C4—H14	1.03 (3)	C15—H115	0.93 (3)
C4—H24	1.03 (3)	C16—C17	1.388 (4)
C5—C6	1.512 (4)	C16—C21	1.394 (3)
C5—H15	0.97 (2)	C17—C18	1.383 (4)
C5—H25	1.03 (3)	C17—H117	1.00 (3)
C6—H16	0.99 (3)	C18—C19	1.376 (4)
C6—H26	0.99 (2)	C18—H118	0.89 (3)
C7—C8	1.524 (4)	C19—C20	1.380 (4)
C7—H17	1.00 (2)	C19—H119	1.00 (3)
C7—H27	1.02 (2)	C20—C21	1.387 (4)
C8—H18	0.99 (3)	C20—H120	0.94 (3)
C8—H28	0.97 (2)	C21—H121	0.96 (3)
C9—C16	1.527 (3)		
			
C7—N2—C4	112.1 (2)	N3—C9—C16	112.77 (19)
C7—N2—C5	108.08 (19)	N3—C9—C10	110.9 (2)
C4—N2—C5	109.3 (2)	C16—C9—C10	110.31 (19)
C8—N3—C6	107.69 (19)	N3—C9—H19	107.4 (12)
C8—N3—C9	111.96 (19)	C16—C9—H19	105.7 (12)
C6—N3—C9	109.63 (19)	C10—C9—H19	109.6 (12)
N1—C1—C2	179.8 (3)	C11—C10—C15	118.4 (3)
C3—C2—C1	119.4 (3)	C11—C10—C9	121.3 (2)
C3—C2—C4	124.6 (3)	C15—C10—C9	120.3 (2)
C1—C2—C4	115.9 (3)	C10—C11—C12	120.6 (3)
C2—C3—H13	115.8 (18)	C10—C11—H111	119.9 (15)
C2—C3—H23	121.7 (16)	C12—C11—H111	119.5 (15)
H13—C3—H23	122 (2)	C13—C12—C11	119.6 (3)
N2—C4—C2	113.3 (2)	C13—C12—H112	124.8 (19)
N2—C4—H14	106.4 (14)	C11—C12—H112	115.6 (19)
C2—C4—H14	108.2 (14)	C14—C13—C12	120.8 (3)
N2—C4—H24	111.9 (14)	C14—C13—H113	119.8 (18)
C2—C4—H24	106.4 (15)	C12—C13—H113	119.4 (18)
H14—C4—H24	111 (2)	C13—C14—C15	119.6 (4)
N2—C5—C6	110.8 (2)	C13—C14—H114	121 (2)
N2—C5—H15	108.4 (13)	C15—C14—H114	119 (2)
C6—C5—H15	110.5 (14)	C14—C15—C10	121.0 (4)
N2—C5—H25	107.7 (15)	C14—C15—H115	120.4 (17)
C6—C5—H25	111.1 (14)	C10—C15—H115	118.5 (17)
H15—C5—H25	108.3 (19)	C17—C16—C21	118.1 (3)
N3—C6—C5	111.1 (2)	C17—C16—C9	121.5 (2)
N3—C6—H16	109.6 (14)	C21—C16—C9	120.3 (2)
C5—C6—H16	111.8 (14)	C18—C17—C16	120.4 (3)
N3—C6—H26	110.7 (12)	C18—C17—H117	120.9 (17)
C5—C6—H26	109.0 (13)	C16—C17—H117	118.7 (17)
H16—C6—H26	105 (2)	C19—C18—C17	121.5 (3)
N2—C7—C8	111.2 (2)	C19—C18—H118	121.9 (19)
N2—C7—H17	110.6 (13)	C17—C18—H118	116.5 (19)
C8—C7—H17	106.9 (13)	C18—C19—C20	118.5 (3)
N2—C7—H27	112.3 (13)	C18—C19—H119	121.8 (18)
C8—C7—H27	108.8 (13)	C20—C19—H119	119.6 (18)
H17—C7—H27	106.8 (19)	C19—C20—C21	120.6 (3)
N3—C8—C7	111.3 (2)	C19—C20—H120	121.2 (17)
N3—C8—H18	109.9 (15)	C21—C20—H120	118.2 (17)
C7—C8—H18	109.5 (14)	C20—C21—C16	120.8 (3)
N3—C8—H28	107.8 (13)	C20—C21—H121	122.3 (16)
C7—C8—H28	108.2 (13)	C16—C21—H121	116.7 (16)
H18—C8—H28	110.2 (19)		
